# Effectiveness outcomes and health related quality of life impact of disease progression in patients with advanced nonsquamous NSCLC treated in real-world community oncology settings: results from a prospective medical record registry study

**DOI:** 10.1186/s12955-017-0735-4

**Published:** 2017-08-14

**Authors:** Mark S. Walker, William Wong, Arliene Ravelo, Paul J. E. Miller, Lee S. Schwartzberg

**Affiliations:** 1 Vector Oncology, 6555 Quince, Suite 400, Memphis, TN 38119 USA; 2 Genentech, Inc., 1 DNA Way, South San Francisco, CA 94080 USA; 3 West Cancer Center, 7945 Wolf River Blvd., Germantown, TN 38138 USA

**Keywords:** Nonsquamous NSCLC, Community oncology, Patient reported outcomes, Treatment effectiveness, Health related quality of life

## Abstract

**Background:**

Treatment options for advanced nonsquamous non-small cell lung cancer (NSCLC) in the first line include platinum-based doublet therapy with or without bevacizumab. This study examined efficacy outcomes and patient reported outcomes (PROs) in a community oncology patient sample.

**Methods:**

Advanced nonsquamous NSCLC patients from 34 U.S. community oncology practices treated in first line with bevacizumab regimens (**A** platinum doublet; gemcitabine doublet; pemetrexed with platinum) or non-bevacizumab regimens (**B** platinum doublet; gemcitabine doublet; **C** pemetrexed with platinum) were recruited for this prospective study. Patient characteristics and clinical outcomes were accessed from routine care records. Three validated and widely used PRO measures of health related quality of life (HRQOL) and symptom burden were collected prospectively at each visit and up to one-year follow-up. Effectiveness outcomes were progression free survival (PFS) and overall survival (OS) assessed by Kaplan-Meier and Cox regression methods. PROs were analyzed with linear mixed model regression to examine changes over time, and the effect of disease progression.

**Results:**

Of 147 patients in the study, 145 provided PRO data. Patients in treatment groups were: A (*n* = 66, 44.9%); B (*n* = 25, 17.0%); C (*n* = 56, 38.1%). A was associated with significantly longer OS than B (HR = 0.341, *p* = 0.0012), and significantly longer than C (HR = 0.602, *p* = 0.0354). PFS results were similar. Irrespective of regimen group and on 12/32 measures, patients showed significant and clinically meaningful worsening of symptoms and HRQOL at disease progression. After disease progression, the pattern of symptom and HRQOL change showed continued worsening.

**Conclusions:**

Bevacizumab-containing regimens were associated with longer PFS and OS compared with non-bevacizumab regimens. PRO measures show disease progression is associated with worsening HRQOL. Delaying disease progression can sustain better HRQL and reduce symptom burden.

## Background

Lung cancer is the leading cause of cancer-related deaths in men and women in the United States, with an estimated 158,080 deaths in 2016 [1]. Approximately 80% of patients with lung cancer present with non-small cell lung cancer (NSCLC) [2]. A majority of these patients have either locally advanced inoperable disease, stage IV metastatic disease, or co-morbid medical conditions that render them unsuitable for surgical intervention [3].

Standard first-line treatments for patients with advanced NSCLC have been platinum-based chemotherapy regimens. Randomized trials have shown that these platinum-based doublet regimens improve survival compared to single agents or best supportive care and are comparably active, producing one-year survival rates of approximately 30 to 40% with a median survival of 8 to 10 months [4–7].

Bevacizumab, one of the earlier targeted agents for NSCLC, was approved by the Food and Drug Administration (FDA) in October 2006 for administration with carboplatin and paclitaxel in unresectable, locally advanced, recurrent or metastatic nonsquamous NSCLC. The Eastern Cooperative Oncology Group (ECOG) Phase III trial (E4599) compared carboplatin paclitaxel with or without bevacizumab in a large cohort of stage IIIB-IV NSCLC patients and included bevacizumab maintenance therapy. Overall survival (OS) was significantly longer for the bevacizumab treated patients (12.3 vs. 10.3 months, HR = 0.79, *p* = 0.003), and the regimen was well tolerated even though there were more bleeding episodes in the bevacizumab group (4.4 vs. 0.7%, *p* < 0.0001) [8].

The role of bevacizumab and its impact on HRQOL has been studied in the context of treatment of nonsquamous NSCLC in clinical trial settings [9, 10]. For example, in the POINTBREAK trial, patients were assessed using the global health related quality of life (HRQOL) Functional Assessment of Cancer Therapy (FACT-G) measure and the FACT-Ntx measure of neurotoxicity. The patient reported outcome (PRO) differences were supported by and consistent with clinician ratings of toxicities [11]. In the AVAPERL trial, global measures of health related quality of life (HRQOL [QLQ-C30]) did not favor either maintenance regimen, but patient role function, fatigue, and appetite loss were better in the bevacizumab only vs. pemetrexed plus bevacizumab maintenance group [12].

POINTBREAK and AVAPERL highlight findings regarding HRQOL in a clinical study setting, and like other clinical trial research examining HRQOL in advanced NSCLC [13, 14], it focused primarily on the effects of treatment. HRQOL research among patients with advanced nonsquamous NSCLC treated in real-world settings has been limited, and what there is has tended to focus on the concordance of patient and physician assessment of symptom severity and patient factors [15]. Additionally, while many clinical studies have begun to use PFS as a primary endpoint, the impact of disease progression on HRQOL is largely unstudied. Some studies have examined the effects of line of therapy and disease state (progressive vs. progression free) [16], but generally have not examined the impact of disease progression itself across different HRQOL domains. The relationship between disease progression and declines in HRQOL has been examined in patients with HER-2 negative metastatic breast cancer in the context of real-world settings [17], but this question has not been addressed in a similar way for patients with NSCLC.

The objective of the current study was to examine treatment effectiveness (PFS, OS) and HRQOL among patients receiving bevacizumab and non-bevacizumab regimens as first-line treatment of advanced, nonsquamous NSCLC in real-world community oncology settings. A secondary objective was to assess the impact of disease progression on HRQOL in this population.

## Methods

### Study design

This was an electronic medical record (EMR) registry study of treatment effectiveness with a prospective component to measure HRQOL via patient reported outcome (PRO) measures in newly diagnosed patients with advanced nonsquamous NSCLC. The design combined prospective with retrospective data collection of key measures. Eligible patients were prospectively identified and consented, and symptom burden and HRQOL measures were prospectively administered over a period of 12 months from the start of first-line treatment for NSCLC. All clinical data were originally collected as part of routine clinical care at the participating sites, and were collected retrospectively as secondary data from the existing EMRs for purposes of this study. This research was reviewed and approved by IntegReview Institutional Review Board, in Austin, Texas.

### Patients and setting

Participating patients were recruited from 34 community oncology practices affiliated with Vector Oncology (Memphis, TN), that agreed to offer the study to their respective patients. Sites were geographically distributed within the U.S. mainland. To be eligible, patients had to meet the following inclusion criteria: stage IIIB or IV nonsquamous NSCLC, no concurrent other malignancy, greater than or equal to 18 years old, and scheduled for first-line therapy with one of the following qualifying treatment regimens.

1. Bevacizumab with either platinum-based doublet, gemcitabine-based doublet, or with pemetrexed and carboplatin (Arm A)
2. Platinum or gemcitabine-based doublet without bevacizumab (Arm B)
3. Pemetrexed with cisplatin or carboplatin (Arm C)

### Procedure

Eligible patients were identified and offered participation by research staff within each participating site. Interested patients provided informed consent. Patients were informed that they would receive incentive gift cards for completion of PRO measures (see below) as they completed various study milestones. Research staff administered the PRO measures every cycle during active treatment and monthly for patients who completed treatment and were in follow-up for up to 1 year. Patients were informed that they could withdraw from the study at any point of their choosing.

After patient consent, all baseline patient demographic and clinical characteristics were abstracted from EMRs using electronic and manual review of the clinical records. Effectiveness outcome data and other clinical variables were also collected retrospectively from the clinical records at the one-year follow-up. Both prospectively collected PRO data and clinical data collected retrospectively at baseline and follow-up were securely transferred from participating sites to the central office of Vector Oncology.

### Study measures

EMR-based study measures included the following: demographic and clinical patient characteristics, treatment regimen and treatment schedule, adverse events, disease progression, and death. Clinical characteristics included performance status, comorbidities, and sites of metastasis at the time of the qualifying diagnosis. Comorbidities included the 16 conditions listed as part of the Charlson Comorbidity Index. We also computed a weighted Comorbidity Index based on presence or absence of the specific conditions as found in the medical records. Disease progression was identified and dated based on physician progress notes and radiological scan records. Adverse events were recorded by system organ class, with specific events noted within system organ class. Clinical data were collected at the time of patient enrollment during cycle 1 or 2 of first-line therapy and at the end of study at 12 months from treatment initiation or death, whichever occurred first.

PRO measures used to measure symptom burden and HRQOL included the following: 1) European Organization for Research and Treatment of Cancer QLQ-C30 version 3.0 (EORTC QLQ-C30) [18] and Lung Cancer Module (QLQ-LC13) [19], 2) Lung Cancer Module of the M.D. Anderson Symptom Inventory (MDASI-LC) [20, 21], and 3) Rotterdam Activity Level Scale (RALS) of the Rotterdam Symptom Checklist (RSCL) [22].

### Statistical methods

General descriptive methods were used to examine demographic and clinical characteristics, and adverse events, with Fisher’s Exact test or Chi-square, and t test or Wilcoxon rank sum test, used to compare patient treatment groups on categorical and continuous variables. Kaplan-Meier survival analysis with log rank test was used for comparison of PFS and OS across treatment groups. Multivariate Cox regression analysis was used to examine PFS and OS, controlling for demographic and clinical covariates.

Linear mixed models were used to assess change in PROs over time, from start of first-line treatment through disease progression, for up to 12 months of follow-up after the start of first-line therapy. Models distinguished first-line from second-line treatment, and slope discontinuity was used to estimate the impact of disease progression. The intercept and the slope for first line and second line were modeled as random. The models also examined treatment group differences, and controlled for demographic and clinical covariates. Methods generally followed those reported by Cnaan et al. and Littell et al. [23, 24]. In general, changes of 5 to 10% of scale range were considered clinically meaningful and represent 0.2 to 0.5 of a standard deviation across the HRQOL measures [25].

## Results

In all, 156 patients were accrued. However, one patient was deemed ineligible after enrollment and eight patients withdrew during the study. The remaining 147 were included in this analysis, of which 145 provided follow-up PRO data.

**Fig. 1:**
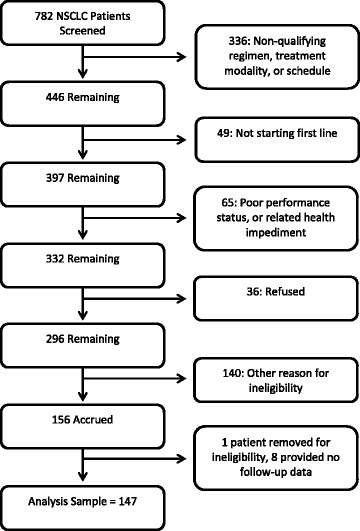
Diagram of Screening and Reasons for Non-Participation in Study

Figure 1 shows the screening process which led to the final sample of 147 patients participating in the study, with 145 providing PRO information. Of 782 patients with a diagnosis of NSCLC, the largest number excluded was 336 due to excluded treatment regimens. Other key exclusions included 49 due to patients already having received first line therapy, 65 due to performance status impairment or other health issues, 36 refused, and 140 for other reasons. One patient was removed from the study after it was discovered the patient was not actually eligible; eight patients who were consented provided no follow up data. Of the 147, 66 (44.9%) received Regimen A, 25 (17.0%) Regimen B, and 56 (38.1%) Regimen C (see Table 1). With a mean age of 64.8 years old, patients were 58.5% male, 86.4% white race. Race and geographic region varied across treatment groups, (*p* = 0.005, *p* = 0.028, respectively), with fewer white patients in Regimen B and fewer patients from the South in Arm C. At the start of first-line treatment, a small proportion of the overall sample (13.6%) had impaired performance status. Stage at initial diagnosis, performance status, rates of baseline brain, bone, and liver metastasis, weighted Comorbidity Index and rate of any comorbid condition did not differ across groups, though there was some nonsignificant variability at the level of individual comorbidities. More than half of patients (55.1%) had at least one comorbidity at the start of first-line treatment. The most common comorbidity was chronic obstructive pulmonary disease (33.3%). The rate at which patients received radiation therapy in the advanced setting varied significantly among treatment groups (*p* = 0.0006), appearing higher in Regimen C (41.1%) compared with A and B (12.1 and 16.0%, respectively). More than 90% of patients had a smoking history.

**Table 1:** Demographic and Clinical Characteristics by Regimen Group

	Regimen A (*N* = 66)	Regimen B (*N* = 25)	Regimen C (*N* = 56)	Total (*n* = 147)
Age, mean (SD)	64.5 (11.11)	67.4 (12.96)	64.1 (9.62)	64.8 (10.90)
Gender				
Male	33 (50.0%)	17 (68.0%)	36 (64.3%)	86 (58.5%)
Female	33 (50.0%)	8 (32.0%)	20 (35.7%)	61 (41.5%)
Race*				
White or Caucasian	57 (86.4%)	17 (68.0%)	53 (94.6%)	127 (86.4%)
Black or African American	6 (9.1%)	6 (24.0%)	2 (3.6%)	14 (9.5%)
Other races	3 (4.5%)	2 (8.0%)	1 (1.8%)	6 (4.1%)
Body Mass Index, mean (SD) (*N* = 146)	26.0 (5.40)	25.4 (5.81)	25.8 (4.37)	25.8 (5.07)
Geographic Region*				
Midwest	10 (15.2%)	7 (28.0%)	16 (28.6%)	33 (22.4%)
Northeast	12 (18.2%)	0 (0.0%)	8 (14.3%)	20 (13.6%)
South	38 (57.6%)	18 (72.0%)	25 (44.6%)	81 (55.1%)
West	6 (9.1%)	0 (0.0%)	7 (12.5%)	13 (8.8%)
Stage at Initial Diagnosis				
I	5 (7.5%)	1 (4.0%)	3 (5.4%)	9 (6.1%)
II	2 (3.0%)	0 (0.0%)	2 (3.6%)	4 (2.7%)
III	4 (6.0%)	3 (12.0%)	5 (9.0%)	12 (8.1%)
IV	52 (78.8%)	21 (84.0%)	46 (82.1%)	119 (81.0%)
Unknown/Not documented	3 (4.5%)	0 (0.0%)	0 (0.0%)	3 (2.0%)
Performance Status (ECOG 2+ or equivalent) ^a^				
Impaired	6 (9.1%)	5 (20.0%)	9 (16.1%)	20 (13.6%)
Not impaired, or not indicated as impaired	60 (90.9%)	20 (80.0%)	47 (83.9%)	127 (86.4%)
Sites of Metastatic Disease at Baseline				
Bone	29 (43.9%)	8 (32.0%)	20 (35.7%)	57 (38.8%)
Brain	11 (16.7%)	4 (16.0%)	17 (30.4%)	32 (21.8%)
Liver	12 (18.2%)	3 (12.0%)	10 (17.9%)	25 (17.0%)
Comorbidity^b^				
Any	32 (48.5%)	14 (56.0%)	35 (62.5%)	81 (55.1%)
Alzheimer’s or other dementia	0 (0.0%)	1 (4.0%)	0 (0.0%)	1 (0.7%)
Cerebrovascular accident	3 (4.5%)	2 (8.0%)	7 (12.5%)	12 (8.2%)
COPD	20 (30.3%)	10 (40.0%)	19 (33.9%)	49 (33.3%)
Cirrhosis	2 (3.0%)	1 (4.0%)	0 (0.0%)	3 (2.0%)
Congestive heart failure	2 (3.0%)	1 (4.0%)	3 (5.4%)	6 (4.1%)
Connective tissue disease	4 (6.1%)	0 (0.0%)	3 (5.4%)	7 (4.8%)
Diabetes	4 (6.1%)	4 (16.0%)	13 (23.2%)	21 (14.3%)
Metastatic solid tumor	1 (1.5%)	2 (8.0%)	1 (1.8%)	4 (2.7%)
Myocardial infarction	2 (3.0%)	1 (4.0%)	4 (7.1%)	7 (4.8%)
Peripheral vascular disease	4 (6.1%)	2 (8.0%)	6 (10.7%)	12 (8.2%)
Renal disease	5 (7.6%)	2 (8.0%)	2 (3.6%)	9 (6.1%)
Ulcer disease	0 (0.0%)	1 (4.0%)	2 (3.6%)	3 (2.0%)
Weighted Comorbidity Index (Mean ± SD)	0.9 ± 1.35	1.6 ± 2.53	1.2 ± 1.44	1.1 ± 1.65
Radiation Therapy in Advanced Setting*	8 (12.1%)	4 (16.0%)	23 (41.1%)	35 (23.8%)
Smoking Status				
Current	11 (16.7%)	6 (24.0%)	15 (26.8%)	32 (21.8%)
Past	50 (75.8%)	17 (68.0%)	36 (64.3%)	103 (70.1%)
Never smoked	4 (6.1%)	1 (4.0%)	5 (8.9%)	10 (6.8%)
Unknown	1 (1.5%)	1 (4.0%)	0 (0.0%)	2 (1.4%)

*Groups differ significantly at *p* < 0.05

^a^ECOG 2+ status, or text record of impairment consistent with ECOG 2+ status. Not impaired defined as ECOG 0 – 1, or if no observed ECOG rating, text indication of nonimpairment, or absence of text indication of impairment

^b^All 16 conditions assessed by Charlson Comorbidity Index. Conditions not present in the sample are not listed

### Effectiveness outcomes

**Fig. 2:**
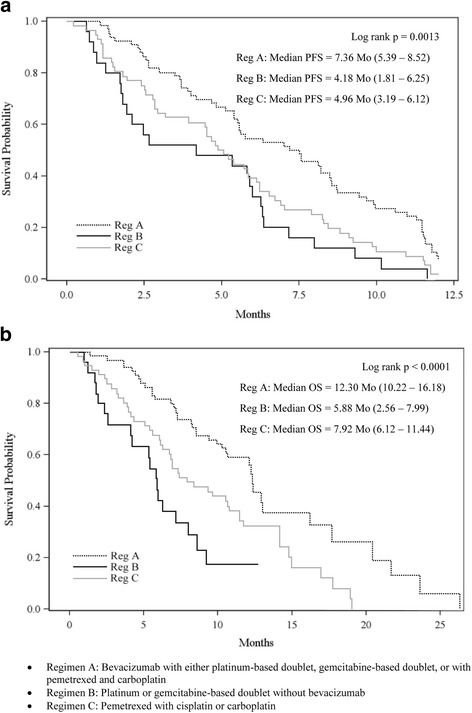
**a** Progression Free Survival in First-Line NSCLC, by Treatment Regimen. **b** Overall Survival in First-Line NSCLC, by Treatment Regimen

Median PFS in first line was 5.59 months overall, with 141 events in 147 patients. Unadjusted median PFS varied significantly by treatment group (Regimen A: 7.36 months, Regimen B: 4.18, Regimen C: 4.96, *p* = 0.0013) (see Fig. 2a). Cox regression analysis of PFS, controlling for demographic and clinical covariates, showed significant variability in PFS by treatment regimen, *p* = 0.0115 (see Table 2). Pairwise comparisons showed that Regimen A was associated with significantly longer PFS than Regimen B (HR = 0.456, *p* = 0.0035), and Regimen A tended to be associated with longer PFS than Regimen C (HR = 0.701, *p* = 0.1070). Impaired performance status was associated with shorter PFS (HR = 2.463, *p* = 0.0011). Other covariates were nonsignificant in predicting PFS.

**Table 2:** Cox Regression of PFS and OS from Start of First-Line Treatment by Treatment Regimen Group

	HR	HR 95% CI	*P* Value
Progression Free Survival Parameters			
Treatment Group (*N* = 147)*			0.0115
Reg B (*N* = 25) vs. Reg A (*N* = 66)	2.191	[1.294 - 3.710]	0.0035
Reg C (*N* = 56) vs. Reg A (*N* = 66)	1.426	[0.926 - 2.197]	0.1070
Age	0.991	[0.973 - 1.009]	0.3071
Gender = Female (vs. Male)	0.990	[0.690 - 1.421]	0.9585
Race Category = Minority (vs. White)	0.796	[0.456 - 1.389]	0.4224
Body Mass Index	0.972	[0.935 - 1.010]	0.1517
Geographic Region = South (vs. Non-South)	1.116	[0.747 - 1.668]	0.5927
Insurance = Any Private (vs. No Private Insurance)	0.909	[0.611 - 1.352]	0.6383
Known Stage IV at Diagnosis (vs. Early or Unknown Stage)	1.332	[0.815 - 2.179]	0.2530
Performance Status = Impaired (vs. Unimpaired)	2.463	[1.431 - 4.239]	0.0011
Brain Mets Present at Diagnosis (vs. Not)	1.111	[0.690 - 1.790]	0.6649
Receipt of Radiation Therapy in the Advanced Setting = Yes (vs. No)	1.207	[0.726 - 2.005]	0.4685
Smoking Status = Current (vs. Former/Never)	0.847	[0.539 - 1.330]	0.4710
Weighted Comorbidity Index	1.038	[0.926 - 1.163]	0.5202
Overall Survival Analysis Parameters			
Treatment Group (*N* = 147)*			0.0040
Reg B (*N* = 25) vs. Reg A (*N* = 66)	2.935	[1.528 - 5.635]	0.0012
Reg C (*N* = 56) vs. Reg A (*N* = 66)	1.661	[1.035 - 2.665]	0.0354
Age	0.995	[0.974 - 1.016]	0.6200
Gender = Female (vs. Male)	0.641	[0.414 - 0.994]	0.0468
Race Category = Minority (vs. White)	0.889	[0.490 - 1.613]	0.6998
Body Mass Index	0.966	[0.924 - 1.010]	0.1276
Known Stage IV at Diagnosis (vs. Early or Unknown Stage)	2.623	[1.422 - 4.838]	0.0020
Performance Status = Impaired (vs. Unimpaired)	2.314	[1.331 - 4.024]	0.0029
Brain Mets Present at Diagnosis (vs. Not)	1.343	[0.832 - 2.169]	0.2277
Weighted Comorbidity Index	1.142	[1.008 - 1.293]	0.0369

*Treatment group effects are reported in text as A vs. comparators (1/HR), but in this table with A as reference condition

As measured from the start of first-line treatment, median OS was 9.67 months overall, with 102 events observed. Unadjusted OS varied significantly by treatment group (Regimen A: 12.30 months, Regimen B: 5.88, Regimen C: 7.92), *p* < 0.0001 (see Fig. 2b). Cox regression analysis of OS, controlling for demographic and clinical covariates, showed significant variability in OS by treatment regimen, *p* = 0.0040 (see Table 2). Pairwise comparisons showed that Regimen A was associated with significantly longer OS than Regimen B (HR = 0.341, *p* = 0.0012), and Regimen A was also associated with significantly longer OS than Regimen C (HR = 0.602, *p* = 0.0354). Male gender, stage IV at diagnosis, impaired performance status, and higher comorbid disease burden were associated with shorter OS.

### Treatment regimen effects on adverse events and patient reported outcomes

Ninety-three patients (63.3%) had at least one adverse event, with no significant differences between regimen groups in the rate at which patients had an adverse event (Regimen A: 71.2%, Regimen B: 56.0%, Regimen C: 57.1%), *p* = 0.1955. Regimen groups did not differ in AE rates at the level of system organ class, except for musculoskeletal events (Regimen A: 0%, Regimen B: 8%, Regimen C: 5.4%, *p* = 0.042).

PRO analysis was based on 145 patients, with 1100 individual PRO surveys. Fifty-nine patients provided PRO data on or after the date of disease progression, though it should be noted that only 75 patients total received therapy beyond first line. The number of patients providing PRO data by Arm included Regimen A: 64 patients (495 surveys) before progression, 27 patients (97 surveys) after; Regimen B: 24 patients (105 surveys) before, 7 patients (14 surveys) after; and Regimen C: 56 patients (313 surveys) before, 25 patients (76 surveys) after. Differences in survey number by group were a function of group sample size and treatment duration, with surveys administered on average every 27 to 29 days during first line. Results from the linear mixed models generally showed no differences in PROs at baseline or over time between treatment regimen groups.

### Disease progression and patient reported outcomes

**Fig. 3:**
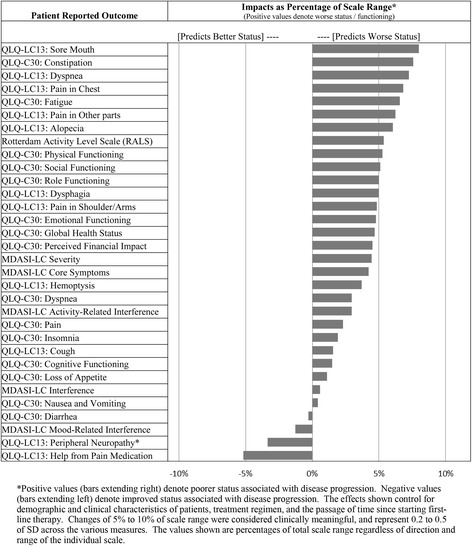
Linear Mixed Model-Implied Effects of Disease Progression and Other Key Predictors

Disease progression had a significant effect on PROs. In particular, many of the PRO endpoints showed deterioration at the time of and generally following disease progression. Modeled as a time varying covariate, disease progression was a statistically significant (*p* < 0.05) or near significant (*p* < 0.10) predictor of adverse impact for 18 of 32 PRO endpoints. Fig. 3 lists each of the PRO endpoints and the percentage deterioration attributable to disease progression for each. Controlling for patient characteristics, treatment regimen, and the passage of time, disease progression had a statistically significant effect on 12 PRO measures (Sore Mouth, Constipation, Dyspnea, Pain in Chest, Fatigue, Pain in Other Parts, Alopecia, RALS Activity Level, Physical Functioning, Social Functioning, Role Functioning, Dysphagia). The average associated deterioration was 5.7% of the instrument range, where changes of 5 to 10% of scale range were considered clinically meaningful, and represented 0.2 to 0.5 standard deviations across the measures. This range of values would be considered to reflect small to moderate clinically meaningful effects if they were between-group differences, but here, as representing longitudinal (within-subject) effects, some of these would be viewed as moderate to large clinically meaningful levels of change [25]. The largest negative effects associated with disease progression were for QLQ-LC13: Sore Mouth (8.0%), QLQ-C30: Constipation (7.6%), and QLQ-LC13: Dyspnea (7.3%). The adverse impact of disease progression on the QLQ-C30: Global Health Status scale was 4.7% (*p* = 0.047), and on QLQ-C30: Physical Functioning scale was 5.3%.

**Fig. 4:**
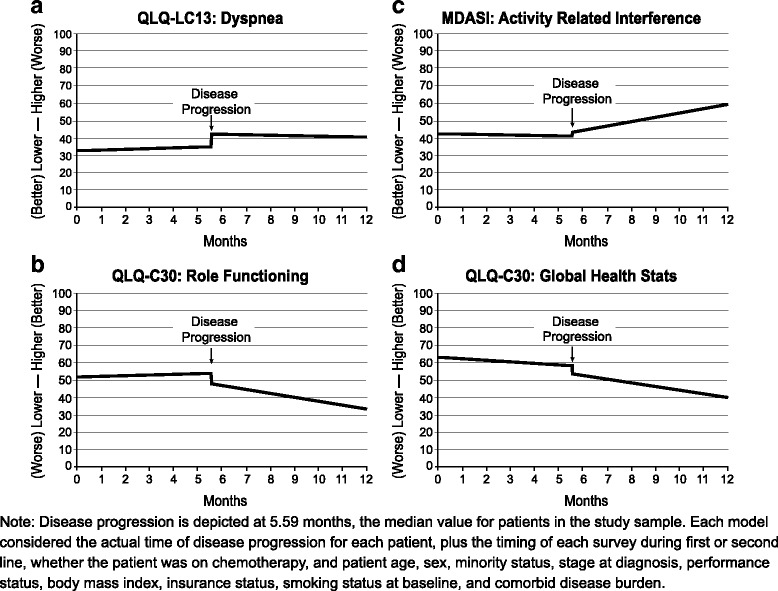
Selected Patient Reported Outcomes from Start of First-Line Therapy, with Model-Implied Impact of Disease Progression

As illustrated in Fig. 4a–d, the combined impact of time and disease progression varied for different PRO measures. Disease progression within the figures is depicted at the median value for patients in the study sample. Models controlled for patient characteristics, treatment regimen, and the timing of each survey during first or second-line treatment. For Dyspnea, a discrete adverse impact of disease progression was observed at the time of the event, but scores were stable before and after progression (Fig. 4a). For Role Functioning (QLQ-C30) and Activity-Related Interference (MDASI), scores were stable pre-progression, with loss of functioning occurring at progression. Further gradual deterioration following progression was also observed (Fig. 4b and c). For Global Health Status (QLQ-C30), patients’ health status appeared to decline gradually over time, punctuated by the effect of the progression event (Fig. 4d). The figures therefore illustrate the different patterns in which cumulative deterioration may occur over time and at disease progression. In contrast, the deterioration due to disease progression as shown in Fig. 3 does not include effects of deterioration that occurred over time either before or after the progression event itself.

Effects of treatment regimen group on PROs were also examined. However, PRO outcomes did not vary significantly by treatment regimen group.

### Patient level predictors of patient reported outcomes

Linear mixed model analyses that included patient level covariates showed that impaired performance status was strongly associated with poorer HRQOL. The largest adverse effects were for RALS (19.4% of instrument range), MDASI-LC Mood-Related Interference (16.7%), and QLQ-C30: Social Functioning (16.0%). In contrast, having private insurance was associated with relative advantage at a level that was significant on 16 measures. The largest positive effects were for QLQ-C30: Perceived Financial Impact (17.9% of instrument range), MDASI-LC Activity-Related Interference (12.3%), and QLQ-C30: Dyspnea (12.1%). The beneficial effect of having private insurance on QLQ-C30: Global Health Status was 10.4% of the instrument range (*p* = 0.0003).

## Discussion

This study examined treatment effectiveness and HRQOL in patients with newly diagnosed advanced nonsquamous NSCLC. Results showed that bevacizumab-containing regimens had better effectiveness outcomes than non-bevacizumab-containing regimens. Results also showed that disease progression was associated with clinically meaningful and statistically significant adverse impact for many HRQOL endpoints. This study complements other published studies that compare effectiveness outcomes in these regimens, here through 1 year of follow-up in real-world community oncology settings.

Compared to bevacizumab-containing regimens (**A** platinum doublet; gemcitabine doublet; pemetrexed with platinum), non-bevacizumab-containing regimens (**B** platinum or gemcitabine doublets; **C** pemetrexed with platinum) were each associated with shorter OS in multivariate adjusted survival analyses. A similar pattern was evident in PFS results, but the A vs. C comparison was not statistically significant. Rate of adverse events was nominally higher in the bevacizumab-containing regimens, but the AE rates among the three regimens were not significantly different. Moreover, extensive longitudinal assessment of HRQOL and symptom burden using PROs did not indicate significant differences between regimen groups.

As assessed by multiple measures repeated over time, patients did experience significant worsening of symptoms and HRQOL at disease progression following first-line treatment of advanced NSCLC. Based on these PRO measures of symptoms and HRQOL, significant effects of disease progression were associated with average adverse impact that was clinically meaningful (minimum change of 5% of scale range). Adverse impact that reached a 5% threshold of clinical meaningfulness was reached in 12 of the 32 PRO-based measures, and as shown in Fig. 3, nearly all the remaining effects were adverse, though of smaller magnitude. The detrimental effects of disease progression were small to moderate as group average effects, but as noted earlier should be assumed to have heightened relevance at the individual patient level [25]. Effects that are moderate on average certainly imply that some patients may experience large and pronounced effects.

The absence of significant differences in HRQOL among treatment regimen groups was not expected. However, this may have been a result of our choice of statistical model rather than a consequence of a complete lack of a treatment effect. Specifically, the study showed significant or near significant effects of treatment regimen on PFS, and, separately, analysis of HRQOL showed that disease progression had a significant adverse impact on many PRO endpoints. If delay of disease progression were the primary way in which a treatment produced a HRQOL benefit, then models that controlled for occurrence of disease progression, as ours did, might show no treatment regimen group effects.

In the clinical setting, providers may wish to pay particular attention to patient-reported symptom burden when there is a disease progression. As described in this report, the maintenance of HRQOL in the PFS state and the adverse effects surrounding disease progression highlight the importance and significance of delaying disease progression as a means to maintain better HRQOL, and to reduce symptom burden in patients with advanced NSCLC.

This research has a number of limitations. The study included only 3 regimen types. Because of this, findings may not generalize to the broader treated population of advanced nonsquamous NSCLC patients. As a non-randomized observational study by design, there may be potential unmeasured confounding variables that could have affected the clinical effectiveness outcomes observed. In addition, these findings are limited to community oncology patients and cannot be assumed to generalize to other settings such as academic and hospital based major research centers. The study also had a number of strengths, including longitudinal assessment of PROs, and assessment of HRQOL across multiple domains.

This research demonstrated improved effectiveness outcomes, including significantly longer OS, associated with bevacizumab-containing treatment regimens in first-line advanced nonsquamous NSCLC. These benefits were observed without significant associated adverse events and without any measurable negative impact on quality of life. Indeed, given study findings of adverse impact of disease progression in general, the improved effectiveness outcomes of bevacizumab-containing therapies may potentially be viewed as conferring HRQOL benefits.

## Conclusions

This research showed improved effectiveness outcomes, including significantly longer OS, associated with bevacizumab-containing treatment regimens in first-line advanced nonsquamous NSCLC. These benefits were observed without significant associated adverse events and without any measurable negative impact on quality of life. The study also showed significant adverse impact of disease progression in general. Given these findings, improved effectiveness outcomes, whether due to chosen therapies or differences in patient management, may potentially be viewed as conferring HRQOL benefits.

## Abbreviations

ECOG: Eastern Cooperative Oncology Group
EMR: Electronic medical record
EORTC: European Organization for Research and Treatment of Cancer
FACT-G: Functional assessment of cancer therapy: general
FACT-Ntx: Functional assessment of cancer therapy: neurotoxicity
FDA: Food and drug administration
HR: Hazard ratio
HRQOL: Health related quality of life
MDASI-LC: Lung Cancer Module of the M.D. Anderson Symptom Inventory
NSCLC: Non-small cell lung cancer
OS: Overall survival
PFS: Progression free survival
PRO: Patient reported outcome
RALS: Rotterdam activity level scale
RSCL: Rotterdam symptom checklist
